# Self-Selected Motivational Music Enhances Physical Performance in Normoxia and Hypoxia in Young Healthy Males

**DOI:** 10.3389/fpsyg.2021.787496

**Published:** 2021-12-10

**Authors:** Kate O’Keeffe, Jacob Dean, Simon Hodder, Alex Lloyd

**Affiliations:** Environmental Ergonomics Research Centre, Loughborough University, Loughborough, United Kingdom

**Keywords:** music, hypoxia, physical performance, motivation, combined stressors

## Abstract

Humans exposed to hypoxia are susceptible to physiological and psychological impairment. Music has ergogenic effects through enhancing psychological factors such as mood, emotion, and cognition. This study aimed to investigate music as a tool for mitigating the performance decrements observed in hypoxia. Thirteen males (mean ± SD; 24 ± 4 years) completed one familiarization session and four experimental trials; (1) normoxia (sea level, 0.209 FiO_2_) and no music; (2) normoxia (0.209 FiO_2_) with music; (3) normobaric hypoxia (∼3800 m, 0.13 FiO_2_) and no music; and (4) normobaric hypoxia (0.13 FiO_2_) with music. Experimental trials were completed at 21°C with 50% relative humidity. Music was self-selected prior to the familiarization session. Each experimental trial included a 15-min time trial on an arm bike, followed by a 60-s isometric maximal voluntary contraction (MVC) of the biceps brachii. Supramaximal nerve stimulation quantified central and peripheral fatigue with voluntary activation (VA%) calculated using the doublet interpolation method. Average power output (W) was reduced with a main effect of hypoxia (*p* = 0.02) and significantly increased with a main effect of music (*p* = 0.001). When combined the interaction was additive (*p* = 0.87). Average MVC force (N) was reduced in hypoxia (*p* = 0.03) but VA% of the biceps brachii was increased with music (*p* = 0.02). Music reduced subjective scores of mental effort, breathing discomfort, and arm discomfort in hypoxia (*p* < 0.001). Music increased maximal physical exertion through enhancing neural drive and diminishing detrimental mental processes, enhancing performance in normoxia (6.3%) and hypoxia (6.4%).

## Introduction

Humans performing in hypoxia are susceptible to debilitating psychological alterations which include severe mood changes, mental fatigue, and neuropsychological impairments (Abraini et al., 1998; Lowe et al., 2007; Stavrou et al., 2015; Martin et al., 2019). Furthermore, it is well established that decrements in both cognitive (Taylor et al., 2016; McMorris et al., 2017; Williams et al., 2019) and physical (Fulco et al., 1998; Goodall et al., 2012; Lloyd et al., 2016) performance are characteristic of hypoxia. These reductions can be largely attributed to the progressive reduction in the partial pressure of oxygen in the alveolar air, arterial blood and ultimately muscle and cerebral tissue due to the reduction in atmospheric partial pressure (PO_2_) on ascent. Cardiac output is increased in an attempt to maintain oxygen saturation, however, combined with the lack of oxygen to the brain, and the need for greater percentages of maximum oxygen uptake (VO_2max_) to maintain a given workload, increases in central and peripheral fatigue are apparent (Amann and Calbet, 2008; Goodall et al., 2012; Lloyd et al., 2016).

Previous research investigating mechanisms which aid in mitigating the detrimental effects of hypoxia on performance have focused largely on physiological mechanisms such as dietary nitrate supplementation (Vanhatalo et al., 2011; Muggeridge et al., 2013), inspiratory muscle training (Downey et al., 2007), and acclimatization strategies (Robertson et al., 2010; Humberstone-Gough et al., 2013). Whilst these interventions have been shown to improve physical and cognitive performance, limited research has investigated the potential impact of psychological interventions to aid in mitigating performance decrements, despite psychological impairment being characteristic of hypoxia (Bahrke and Shukitt-Hale, 1993; Virués-Ortega et al., 2004). Psychological constructs which have been investigated in hypoxia include mood (Lane et al., 2004; Virués-Ortega et al., 2006), anxiety (Fagenholz et al., 2007; Feldman et al., 2013; Boos et al., 2018), and personality (Bahrke and Shukitt-Hale, 1993). Indeed, it is well established that these psychological constructs independently incur detrimental impacts on physical and cognitive performance in normoxia (Raglin, 2001; Woodman and Hardy, 2003; Lane et al., 2009).

Research investigating the use of psychological strategies as interventions to help alleviate the performance decrements observed in extreme environments has focused mainly on observations in the heat. Research has incorporated psychological strategies such as; (1) motivational self-talk which was found to increase power output during a 30-min cycling trial at a fixed rating of perceived exertion in the heat (35°C) (Hatzigeorgiadis et al., 2018); (2) self-selected motivational music which was shown to increase total work over a 15-min cycling time trial by ∼10% (36°C) (English et al., 2019); and (3) a package of psychological skills training incorporating goal setting, arousal regulation, mental imagery, and positive self-talk which was seen to improve running performance by increasing the distance covered during a maximal-effort run by 8% in the heat (30°C) (Barwood et al., 2008). Hence, this research demonstrates the positive impact of psychological strategies to aid in mitigating performance decrements observed in the heat, warranting further investigation into the effectiveness of such strategies to similarly reduce the decrements observed in hypoxia.

Motivational music has been shown to have ergogenic effects on physical and cognitive performance through enhancing mood, arousal, and cognitive processing (Mammarella et al., 2007; Särkämö et al., 2013), and further, increasing power output (Karageorghis and Priest, 2012a), strength endurance (Crust and Clough, 2006), and improving oxygen utilization (Terry et al., 2019). Despite the known impact of motivational music as a facilitator of performance in normoxia and heat, the impact of self-selected motivational music on performance in hypoxia has not been investigated. Given the effect of hypoxia on cerebral oxygenation and the potential for neuropsychological limitations on exercise performance (Lowe et al., 2007), it is possible that motivational music’s impact on performance is altered by the presence of hypoxia. Alternatively, if the impact of hypoxia on performance is primarily physiological in origin, the impact of music in hypoxia should be similar to that of normoxia (Lloyd et al., 2015). Another potential outcome is that, in cases where hypoxia becomes very severe, it is possible that the hypoxic stressor abolishes the ergogenic benefits of music due to hypoxia likely taking precedence as the dominant factor impacting performance (Lloyd et al., 2016; Bradbury et al., 2019).

To understand the combined nature of hypoxia and music on exercise performance, the present study adopted an individual and combined factors approach. When two individual stressors are combined, three outcomes are possible. These are: additive, antagonistic, and synergistic. These outcomes have been discussed in detail with examples by Côté et al. (2016) and Lloyd and Havenith (2016). Briefly, additive effects occur when the combined effect of two stressors is equal to the sum of the absolute impact of each stressor individually. This is categorized as a non-interactive state using analysis of variance (ANOVA) statistics. Alternatively, interactions (synergism and antagonism) are observed when the effect of the combined stress is greater, or smaller, than the sum of the effect of each stressor individually. Interactive states result in a significant interaction statistic using ANOVA.

In this study, one stressor (hypoxia) and one facilitator (music) were combined. Thus, an additive effect occurs when the sum of the positive individual impact of the facilitator (i.e., music) and the negative individual effect of the stressor (hypoxia) are equal to the combined effect, while significant deviations from additive states are considered interactive. On this basis, the current research aimed to test the hypothesis that music impacts both normoxic and hypoxic physical performance equally resulting in an additive (non-interactive) combined effect.

## Materials and Methods

### Participants

Participants comprised of volunteers from Loughborough University. Thirteen healthy males aged 24 ± 4 years (mean ± SD) (weight: 73 ± 11 kg; height: 178 ± 5 cm), that satisfied the minimum physical activity requirement (30-min moderate intensity, physical activity on at least 3 days each week for at least 3 months) with no history of neurological or auditory disability were recruited. Participants were paid for their participation pro rata. The study was approved by the Loughborough University Ethics Committee (R17-P190) and was conducted in compliance with the Declaration of Helsinki except registration in a database (2013).

### Experimental Design

All experiments were conducted in the Environmental Chamber (T.I.S.S. Peak Performance, Series 2009 Climate Chambers) at the Environmental Ergonomics Research Centre, Loughborough University, United Kingdom. Participants were requested to come in at the same time of day for each trial in a similar physical state as the previous trial. Participants were also requested not to consume any food or caffeine for up to 2-h prior to participation in the study. Trial order was counterbalanced with a minimum of 48-h between trials. In total, the current study consisted of five trials, in which four main experimental trials followed an initial familiarization session.

The present study incorporated the use of self-selected motivational music. Prior to attending the familiarization session, participants were asked to self-select their top 10 motivational songs. They were asked to choose songs that would motivate them during exercise where “motivate” meant “make you want to exercise harder and/or longer” (Karageorghis et al., 2006). In the familiarization session participants rated their chosen songs using the Brunel Music Rating Inventory-2 (BMRI-2). This inventory rates music based on the rhythm, style, melody, tempo, sound, and beat of the music on an agreement scale from 1 (strongly disagree) to 7 (strongly agree). The top seven rated songs were chosen as the most motivational for each individual participant and subsequently used in the main experimental trials.

Following the music selection protocol, participants were accustomed to the experimental procedures and a full rehearsal of the physical performance test was completed. The physical performance test included a time trial, as well as a series of brief and sustained isometric neuromuscular tests, including supramaximal nerve stimulation. For full details of the physical performance protocol refer to section: “*2.4 Physical Performance Protocol*.” This concluded the familiarization session.

### Experimental Overview

The present study consisted of four [FiO_2_ (fraction of inspired oxygen) × 2 and music; ×2] main experimental trials. The trials included: (1) normoxia (sea level, 0.209 FiO_2_) and no music; (2) normoxia (0.209 FiO_2_) with music; (3) normobaric hypoxia (∼3800 m, 0.13 FiO_2_) and no music; and (4) normobaric hypoxia (0.13 FiO_2_) with music. Normobaric hypoxia was manipulated using the inbuilt hypoxic air generator in the environmental chamber (T.I.S.S. Peak Performance, Series 2009 Climate Chambers). A Servomex gas analyzer (570A, Sussex, United Kingdom) was used to monitor the oxygen level inside the chamber, as well as the inbuilt analyzer on the chamber.

**FIGURE 1:**
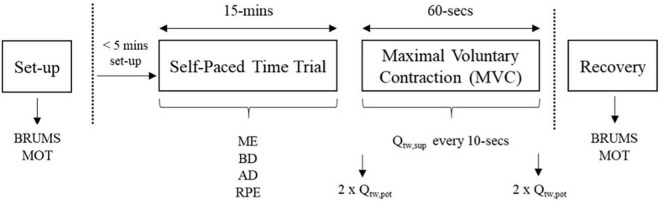
Schematic of main trials. BRUMS, brunel mood scale; MOT, motivation scale; ME, mental effort, Borg CR-100; BD, breathing discomfort, Borg CR-10; AD, arm discomfort, Borg CR-10; RPE, rating of perceived exertion, Borg 6-20 scale; MVC, maximal voluntary contraction; Q_tw,pot_, resting/potentiated doublet; Q_tw,sup_, superimposed doublet.

All experimental trials were completed at 21°C with 50% relative humidity. In each of the main experimental trials, participants completed a protocol (Figure 1) that consisted of: (1) 15-min time trial on an arm-bike, followed by (2) a 60-s isometric maximal voluntary contraction (MVC) of the biceps brachii with supramaximal nerve stimulation. Participants were blinded to the environmental condition.

### Physical Performance Protocol

All exercise testing was conducted on a custom-made arm-bike rig, built by the Environmental Ergonomics Research Centre. The device was developed to measure arm bike performance and facilitate a fast transition to measure neuromuscular function of the biceps brachii. Therefore, the device consisted of a seat and chest harness, a hand bike ergometer (Lode Angio B.V., Groningen, The Netherlands) located in front of the participant, and on the side, a right arm support which included an adjustable force transducer and wrist strap (O’Keeffe et al., 2021).

In each experimental condition, upon entering the chamber, participants practiced a series of 3-s MVC’s with supramaximal nerve stimulation. Participants then conducted a 15-min time trial on the arm bike where they were instructed to perform as much work as possible. Participants did not receive any external reinforcement for the duration of the physical performance protocol and were only informed when they had 5-min remaining in the time trial. This mode of exercise performance was used to represent self-regulated upper body work which is characteristic of high-altitude pursuits. Upper body work is particularly dominant in climbing sections, with scrambling (ascending steep terrain using hands), technical climbing using ropes, hand holds, ladders, ice axes and carrying, pushing, and pulling loads all prominent features. In addition, a time trial performance component is representative of the self-regulated work and further, the arm bike ergometer was used, as the speed of transition from the ergometer to the custom-built rig for performance of the MVC was important to ensure accuracy of the neuromuscular assessment.

The resistance factor on the arm bike ergometer was determined based on the linear relationship with a traditional monarch brake weight and is defined as; resistance factor alpha = 0.0161 × brake weight + 0.0008. The brake weight corresponds to 1% of a participant’s familiarization body mass (73 ± 11 kg). This particular brake weight was chosen based on pre-experimental piloting and previous research conducted in the laboratory using this method (O’Keeffe et al., 2021). By incorporating this method, performance in the time trial (i.e., power output) is therefore dependent on cadence.

The self-selected motivational music was compiled into playlists for each individual participant and played through two speakers (Mission, United Kingdom) connected to an integrated stereo amplifier (Sony, TA-FE230), placed directly in front of the arm-bike rig (1.2-m from the participants head). Music was played at a standardized noise level of 80-dBA (at participant’s ears) monitored using a sound level meter (Model: LxT 831, Larson Davis Inc., United States) as per previous research indicating that 80-dBA enhances exercise performance (Edworthy and Waring, 2006; Karageorghis et al., 2018). Selected music ranged from slow-paced church music to faster-paced hip hop and heavy metal. The music was played from the start of the 15-min time trial and stopped at the end of the MVC.

Following the 15-min time trial, participants performed a 60-s MVC with simultaneous nerve stimulation every 10-s. Supramaximal nerve stimulation (using the doublet interpolation method) was used to quantify central and peripheral fatigue (Merton, 1954; Herbert and Gandevia, 1999). Pre-and post-exercise, during the MVC of the right bicep brachii, electrical impulses (300V, doublet square-wave, with a 10-ms stimulus interval) were delivered using a nerve stimulator (DS7AH, Digimeter Ltd., United Kingdom). The stimulator anode was placed over the medial border of the right scapula, and the cathode was placed on the distal tendon of the right bicep. The optimal position for stimulation of the biceps brachii was confirmed based on the force output of the biceps in response to submaximal current stimulation (∼50 mA) and then progressively increased by 10 mA until a plateau in the mechanical response of the muscle was observed (91 ± 25 mA).

After securing the right arm in the arm support, two stimulations were first given at rest. Following this, participants performed a 60-s sustained MVC in which superimposed doublet twitches were evoked (Qtw,_sup_) every 10-s. The sustained MVC was immediately followed by two further resting potentiated doublet twitches (Qtw,_pot_) 1-s after full muscle relaxation. Voluntary muscle activation of the bicep (VA%) was calculated using the equation: VA% = (1 − Qtw,_sup_/Qtw,_pot_) × 100 (Folland and Williams, 2007) (where Qtw,_sup_ is the average of doublet twitches evoked over the 60-s MVC).

### Measures

#### Subjective Measures

Participants were familiarized with all subjective measurements in the familiarization session. Subjective scales were used to monitor self-reported scores of mental effort (Borg CR-100 scale), ratings of perceived exertion (RPE) (Borg 6-20 scale), arm discomfort (Borg CR-10 scale), and breathing discomfort (Borg CR-10 scale), which were recorded every 3-min during the physical performance protocol (Borg, 1998). Mood was assessed using the Brunel Mood Scale (BRUMS). Pre- and post-test scores of fatigue, vigor, confusion, depression, tension, and anger were examined (Terry et al., 2003). Motivation was assessed using the task and success motivation scales (Matthews et al., 2013) where motivation was measured pre and post-exercise.

#### Physiological Measures

Physiological measures were recorded from the beginning of the 15-min time trial. Heart rate (HR) was monitored using a Garmin heart rate strap (Model: HRM-DUAL, Garmin, United Kingdom). Oxygen saturation of peripheral blood (SpO_2_) was monitored every minute for participant safety from the beginning of the time trial using a pulse oximeter attached to the left ear lobe (Model: 8500, Nonin, United States).

Oxygen consumption (VO_2_: ml⋅kg^–1^⋅min^–1^) was recorded using a breath-by-breath gas analyzer system (Model: Quark RMR, COSMED, Italy). Prior to each experiment, this analyzer system was calibrated to ensure accurate measurement. The O_2_ and CO_2_ gas analyzers were calibrated before each test using a certified calibration gas. Following this, a flowmeter, which is a bidirectional turbine with a 28-mm diameter, used to detect ventilation rate, was standardized using a 3-L calibration syringe.

Tissue oxygenation of the left bicep was measured using near infrared spectroscopy (NIRS) (Model: moorVMS-NIRS, Moor Instruments, United Kingdom). This non-invasive measure generates a near infrared light that penetrates the human tissue *via* a probe attached to the skin (i.e., in this study on the left bicep), and secured using specific adhesive pads. A standard probe (Model: moorVMS-NIRS probe, Moor Instruments, United Kingdom) includes a detector head and an emitter head, 30-mm apart. The emitter head contains two near infrared LEDs which penetrate the tissue and pass through in a curvilinear fashion (Scheeren et al., 2012) to the detector head which contains two identical photodiodes. An indication of the bicep muscle tissue oxygenation is then provided based on the ability of NIRS to detect oxygenated and deoxygenated hemoglobin through the changes in oxygen dependent light absorption (Lefferts et al., 2016). Absolute concentrations of oxyhemoglobin and deoxyhemoglobin are reported and expressed as percentages. Tissue oxygenation of the bicep was collected *via* a rolling average every 3-min.

#### Physical Performance Measures

The primary physical performance outcome is average power output produced in watts (W) which was recorded *via* a rolling average every 3-min. Peak power output (W) was also recorded. Data was digitally sampled using data acquisition software DASYLab. Maximal voluntary force (N) and average voluntary force (N) was recorded in Newtons (N) over the 60-s MVC.

### Data Analysis

Two-way repeated measures ANOVA was used for the analysis of the main effects and differential impact of music and hypoxia on physical performance (2 × 2: FiO_2_ × music). Within trials, pre-test and post-test were compared using paired sample *t*-tests with Bonferroni correction for multiple comparisons. To calculate the power required to observe a main effect of hypoxia (2 levels) and music (2 levels), a power analysis conducted in G*Power (Faul et al., 2007) incorporating a large effect size (*f* = 0.4), power of 0.8 with an α err prob of 0.05 yielded a sample size of 12. The power required for a one-tailed (pre to post) *t*-test using a large effect size of *d* = 0.8, a power of 0.8, and an α err prob of 0.05 outputted a required sample size of 12. A 95% confidence level was used to test significance (*p* < 0.05). When Mauchly’s Test of Sphericity was significant (*p* < 0.05), the Greenhouse–Geisser adjustment was used. Effect sizes, taken from SPSS, are reported as partial eta squared $\left(\eta_{p}^{2}\right)$ for main effects and interactions. Data is presented as mean ± SD.

## Results

### Physical Performance

**FIGURE 2:**
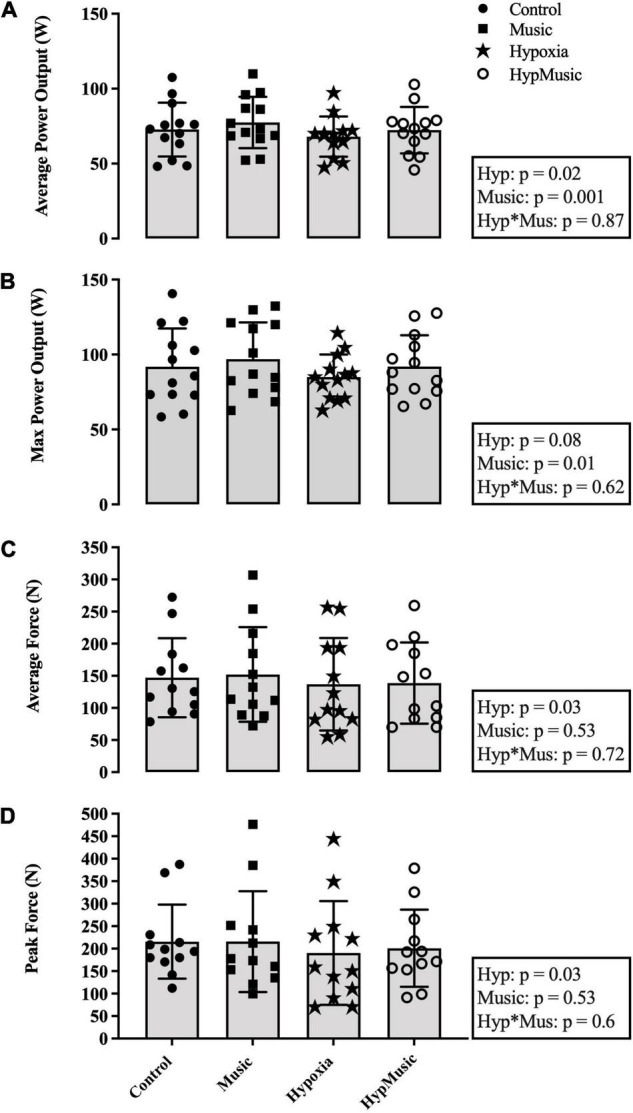
**(A)** Average power output (W). **(B)** Maximum power output (W). **(C)** Maximal voluntary contraction [average force over 60-s (N)]. **(D)** Peak force (N). Main effects and interactions are displayed in the respective boxes where Hyp signifies Hypoxia and Hyp*Mus signifies the Hypoxia × Music Interaction.

**FIGURE 3:**
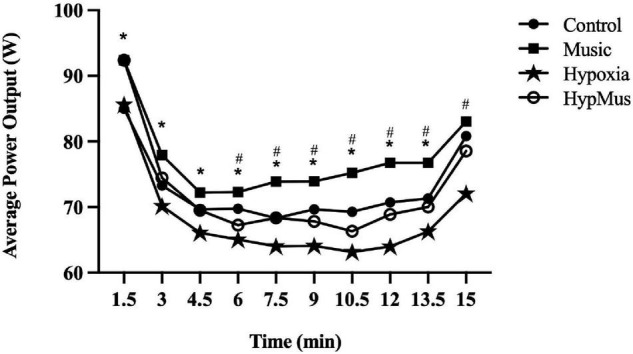
Pacing strategies across the four main experimental trials. *Main effect of music. ^#^Main effect of hypoxia.

Average power output (W) was reduced with a main effect of hypoxia (*p* = 0.02, $\eta_{p}^{2}=0.63$) and significantly increased with a main effect of music (*p* = 0.001, $\eta_{p}^{2}=0.38$) (Figure 2A). When combined the interaction was additive (*p* = 0.87, $\eta_{p}^{2}=0.002$). This indicates that the effect of one factor (e.g., FiO_2_) was not altered by a change in another factor (e.g., music). Maximum power output was unaffected by hypoxia (*p* = 0.08, $\eta_{p}^{2}=0.23$) but was significantly increased with a main effect of music (*p* = 0.01, $\eta_{p}^{2}=0.42$) (Figure 2B). The pacing strategies for each trial are presented in Figure 3. A main effect of music, where music increased average power output was observed from minute 1.5 until minute 13.5, *p* ≤ 0.04. Further, a main effect of hypoxia, where hypoxia decreased average power output was observed from minute 6 until minute 15, *p* ≤ 0.04 (Figure 3).

**FIGURE 4:**
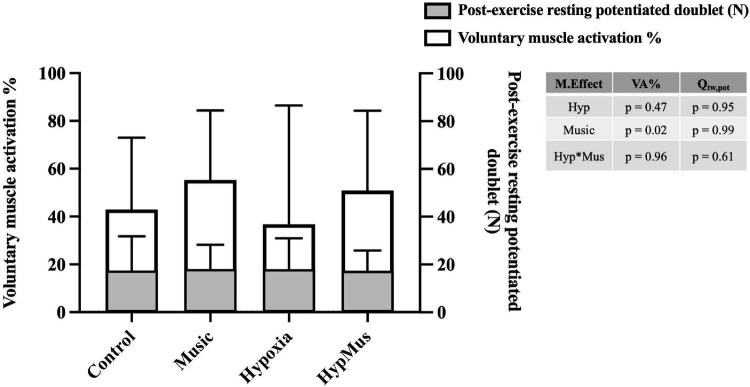
Voluntary muscle activation % represented in the white bars, and post-exercise resting potentiated doublet force (N) represented in the gray bars. Main effects are outlined in the table to the right of the graph, M.Effect, main effect; VA%, voluntary muscle activation %; Qtw,_pot_, post-exercise resting potentiated doublet force; Hyp, hypoxia; Hyp*Mus, hypoxia × music interaction.

One participant was removed from the MVC analysis due to technical difficulties. During the post-exercise MVC, average MVC force (N) was reduced in hypoxia (*p* = 0.03, $\eta_{p}^{2}=0.35$). No effect of music was observed on average MVC force, *p* = 0.53 $\left(\eta_{p}^{2}=0.03\right)$ (Figure 2C). Similarly, peak MVC force (N) was reduced in hypoxia (*p* = 0.03, $\eta_{p}^{2}$ = 0.35) and no effect of music was observed (*p* = 0.53, $\eta_{p}^{2}$ = 0.03) (Figure 2D). No effect of hypoxia was observed on VA% of the bicep brachii, *p* = 0.47 $\left(\eta_{p}^{2}=0.05\right).$ However, a main effect of music, where music increased VA% in both normoxia (55.32 ± 27.82%) and hypoxia (47.87 ± 28.7%), *p* = 0.02 $\left(\eta_{p}^{2}=0.4\right),$ was observed (Figure 4). No interaction was observed indicating an additive effect, *p* = 0.96. Post-exercise resting potentiated doublet twitch force (N) was unaffected by hypoxia (*p* = 0.95) or music independently (*p* = 0.99), with no interaction observed (*p* = 0.61) (Figure 4).

### Physiological Outcomes

Two participants were removed from the analysis of NIRS due to technical errors. A main effect of hypoxia was observed on muscle deoxygenation of the bicep where hypoxia increased muscle deoxygenation (HHb) (*p* = 0.05, $\eta_{p}^{2}=0.34$). Music had no effect on muscle deoxygenation, *p* = 0.40 $\left(\eta_{p}^{2}=0.07\right)$ (Table 1). Furthermore, no effect of hypoxia (*p* = 0.7, $\eta_{p}^{2}=0.02$) or music (*p* = 0.34, $\eta_{p}^{2}=0.09$) was observed on oxygenation of the muscle (O_2_Hb) or systemic (VO_2_) oxygen consumption (*p* ≥ 0.12) (Table 1). No effect of hypoxia (*p* = 0.32), music (*p* = 0.29), or interaction (*p* = 0.51) was observed on heart rate.

**TABLE 1:** Physiological measures of mean heart rate (HR, bpm), oxygen consumption (VO_2_, ml⋅kg^–1^⋅min^–1^), muscle deoxygenation (HHb), and muscle oxygenation (O_2_Hb) across conditions.

Variable	Control	Music	Hypoxia	HypMusic	Main effects
HR	138 ± 19	143 ± 20	142 ± 18	144 ± 25	
VO_2_	23.74 ± 3.8	24.76 ± 3.8	22.78 ± 3.8	23.11 ± 4.89	
Deoxy, HHb	162 ± 31	158 ± 34	174 ± 53	157 ± 43	Hypoxia
Oxy, O_2_Hb	160 ± 37	182 ± 45	141 ± 58	138 ± 35	

*Significant main effects are outlined where p < 0.05.*

### Discomfort and Perceived Effort

**FIGURE 5:**
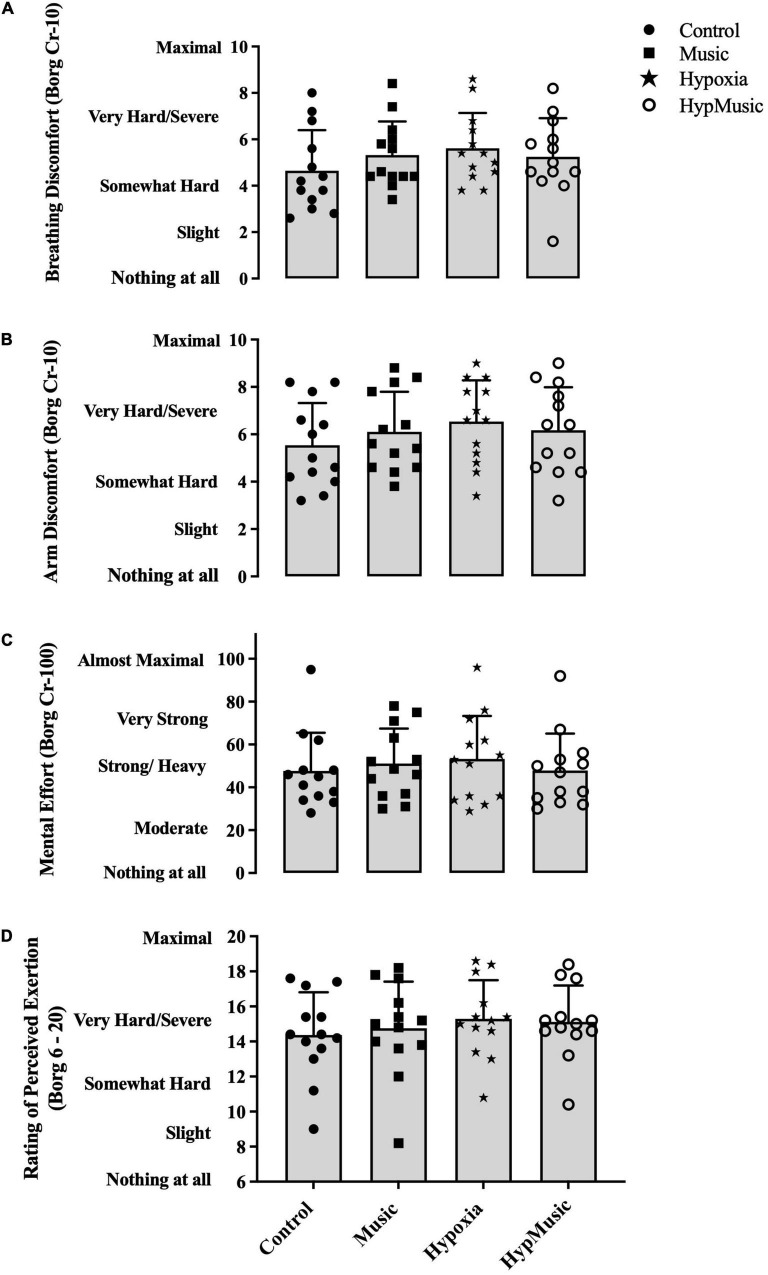
Subjective ratings of **(A)** breathing discomfort (Borg CR-10 scale), **(B)** arm discomfort (Borg CR-10 scale), **(C)** mental effort (Borg CR-100 scale), and **(D)** rating of perceived exertion (Borg 6-20 scale). Data is averaged over the 15-min time trial.

Subjective ratings of discomfort and perceived effort are presented as an average over the 15-min time trial. Breathing (5.6 ± 1.5, $\eta_{p}^{2}=0.14$) and arm discomfort (6.5 ± 1.7, $\eta_{p}^{2}=0.25$) were increased in the presence of hypoxia, *p* ≤ 0.002, but music had no effect on breathing discomfort $\left(\eta_{p}^{2}=0.02\right)$ or arm discomfort $\left(\eta_{p}^{2}=0.02\right),$ *p* ≥ 0.24. However, a significant interaction where music decreased subjective scores of breathing (5.2 ± 1.7, *p* < 0.001, $\eta_{p}^{2}=0.27$) (Figure 5A) and arm discomfort (6.2 ± 1.81, $\eta_{p}^{2}=0.21$) in hypoxia was observed (Figure 5B). Independently, no effect of hypoxia (*p* = 0.41, $\eta_{p}^{2}=0.01$) or music (*p* = 0.36, $\eta_{p}^{2}=0.01$) was observed on subjective scores of mental effort. However, when combined, a significant interaction where music reduced ratings of mental effort was reported (47.84 ± 17.25, *p* = 0.001, $\eta_{p}^{2}=0.16$) (Figure 5C). A main effect of hypoxia was observed on RPE (15 ± 2, *p* < 0.001, $\eta_{p}^{2}=0.21$), where ratings were increased (Figure 5D). However, no effect of music was seen on RPE (*p* = 0.42, $\eta_{p}^{2}=0.01$), and no interaction was observed (*p* = 0.06, $\eta_{p}^{2}$ = 0.05).

### Mood and Motivation

Motivation questionnaires were analyzed in terms of task motivation, success motivation, and overall motivation, and were measured pre-exercise and post-exercise. There were no significant differences in pre-task motivation scores (*p* = 0.32), pre-success motivation scores (*p* = 0.56), or pre-overall motivation scores (*p* = 0.17).

Motivation scores were analyzed in relation to percentage change from pre-test to post-test (Table 2). No effect of music (*p* = 0.30, $\eta_{p}^{2}=0.08$) or hypoxia (*p* = 0.20, $\eta_{p}^{2}=0.13$) was observed on success motivation, with no interaction presented (*p* = 0.68, $\eta_{p}^{2}=0.02$). Further, there was no effect of music (*p* = 0.19, $\eta_{p}^{2}=0.13$), hypoxia (*p* = 0.07, $\eta_{p}^{2}=0.25$), or interaction (*p* = 0.79, $\eta_{p}^{2}=0.01$) on task motivation. Likewise, overall motivation was unaffected by music (*p* = 0.75, $\eta_{p}^{2}=0.01$) and hypoxia (*p* = 0.29, $\eta_{p}^{2}=0.09$), with no interaction (*p* = 0.67, $\eta_{p}^{2}$ = 0.02).

**TABLE 2:** Subjective measures of motivation and mood.

Variable	Control		Music		Hypoxia		HypMusic	
	Pre	Post	Pre	Post	Pre	Post	Pre	Post
Motivation: task	19.2 ± 4.7	19.2 ± 6.3	20.6 ± 4.6	22.3 ± 4.4	19.1 ± 5.9	20.3 ± 5.7	18.9 ± 6.2	21 ± 4.9
Motivation: success	18.9 ± 3.4	19.9 ± 4.3	19.9 ± 4.1	19.6 ± 4.5	19.2 ± 4	20.8 ± 4.5	19.1 ± 3.7	20.3 ± 3.9
Motivation: overall	3 ± 0.9	3.2 ± 0.7	3.4 ± 0.6	3.7 ± 0.4	3.3 ± 0.4	3.4 ± 0.6	3.2 ± 0.8	3.3 ± 0.6
BRUMS: vigor	53.3 ± 14.8	51 ± 13.3	51.5 ± 19.5	61.2 ± 20.5	52.9 ± 11.8	50 ± 23	47.7 ± 20	47.5 ± 14.5
BRUMS: fatigue	17.4 ± 23.7	30 ± 21.3	13.9 ± 10.5	13.9 ± 10.5	20.5 ± 24.6	30.9 ± 22.9	9.2 ± 10.8	20.7 ± 9.2
BRUMS: confusion	15.6 ± 18.4	9.5 ± 12.8	13.5 ± 10.7	6.3 ± 6.7	7.3 ± 7.3	7.5 ± 4.2	15.6 ± 12.3	9.6 ± 6.1
BRUMS: depression	4.5 ± 7.3	3.6 ± 7.5	0	0	5.2 ± 10	4.1 ± 10.2	0.5 ± 1.2	0.1 ± 0.3
BRUMS: tension	7.5 ± 8	3.2 ± 5.1	6.9 ± 6.2	2.2 ± 5	8 ± 6.5	3 ± 5.2	11.7 ± 8.8	2 ± 5.1
BRUMS: anger	4.2 ± 7.4	3.2 ± 7.5	0.1 ± 0.3	0.1 ± 0.3	2.2 ± 5	2.2 ± 5	0.1 ± 0.3	4.2 ± 6.3

*Motivation is divided into task motivation, success motivation, and overall motivation. Mood, measured using the Brunel Mood Scale, includes vigor, fatigue, confusion, depression, tension, and anger. Subjective measures were taken pre and post exercise in each condition. Data is presented as mean ± SD.*

Regarding mood, there were no significant differences in pre-test scores across all moods, *p* ≥ 0.23. Likewise, across trials, there were no main effects of hypoxia (*p* ≥ 0.11) or music (*p* ≥ 0.15) on any post-test subjective mood scores (Table 2).

## Discussion

This study aimed to investigate the individual and combined effects of self-selected motivational music on physical performance in hypoxia. The results demonstrated that hypoxia had a negative impact on average power output in the 15-min time trial and maximal isometric voluntary contraction of the bicep. When music and hypoxia were combined, VA% and average power output were increased with music, compared to the hypoxic trial with no music, demonstrating an additive effect. This indicates that when combined, music can improve performance in normoxia and hypoxia by potentially limiting the onset of central fatigue (i.e., the reduction in VA%). Furthermore, subjective ratings of mental effort, breathing discomfort and arm discomfort were reduced in the presence of music in hypoxia.

### Time Trial Performance

In general, the use of the hand bike ergometer results in lower VO_2_ (ml⋅kg^–1^⋅min^–1^) scores observed largely due to the specificity of the muscle groups that are involved and the lower amount of active muscle tissue as compared to other modes of exercise (e.g., rowing and cycling). The primary working muscles used in this exercise mode are the biceps, triceps, and deltoid (Mitropoulos et al., 2017) which are naturally smaller and less conditioned for endurance exercise. Nevertheless, in the present study, music increased power output over a 15-min time trial in both normoxia and hypoxia. In normoxia, the ergogenic benefits of music on exercise performance have been well established (Karageorghis and Priest, 2012a,b; Terry et al., 2020) and support the finding of the current study. In hypoxia, however, no previous research has been conducted on whether similar ergogenic effects of music on performance can be seen. In general, in the context of extreme environments, research investigating the impact of music on exercise performance is limited. Of this research, English et al. (2019) investigated the impact of music on a 15-min time trial on a bike ergometer in heat stress (36°C). They found that with self-selected motivational music, total work output in the heat was increased by ∼10%. Results from the current study corroborate these findings as, in the presence of hypoxia, exercise performance was increased by 6.4% and similarly in normoxia, exercise performance was increased by 6.3%. Further research is warranted to explore the impact of music as an intervention in other extreme environmental conditions (i.e., cold and rain), and to investigate the differential impact of music in greater levels of hypoxia (i.e., >3800 m), where the decrements in physical, cognitive, and psychological constructs may be exacerbated (Bahrke and Shukitt-Hale, 1993; Goodall et al., 2012; Komiyama et al., 2017).

### Central and Peripheral Fatigue

A unique element of the present study involved participants completing a 60-s isometric voluntary contraction with simultaneous nerve stimulation to quantify central and peripheral fatigue using the doublet interpolation method. In the presence of hypoxia, maximal voluntary force was reduced when compared to the normoxic control. However, when combined, music increased the VA% of the bicep brachii. This is the first study to demonstrate that self-selected motivational music increases VA%, i.e., neural drive to the muscle, mitigating the onset of central fatigue in both normoxia and hypoxia. Limited research investigating the impact of music on muscular endurance exercise exists. One such study investigating the effects of motivational music on a muscular endurance task (isometric weight-holding task with a 1.1 kg dumbbell) observed that participants were able to sustain the task for 11% longer when motivational music was played throughout the task (Crust and Clough, 2006). Another study investigating the effects of music tempo and intensity on grip strength found that fast tempo (126 bpm) and high intensity (80 dBA) music increased grip strength coupled with greater subjective scores for positive affect and arousal (Karageorghis et al., 2018). Whilst these studies indicate the potential for music to improve muscular endurance performance, they did not measure neuromuscular activity and hence, further research is warranted to support the current findings that self-selected motivational music increases VA% in normoxia and hypoxia.

### Mechanisms of Music

Significant advances have been made toward understanding the mechanisms underpinning the effects of music on exercise performance (Terry et al., 2020). Rejeski’s (1985) model of parallel information processing has been widely used and accepted as an explanation to how music impacts exercise performance. In this model, afferent feedback to the central nervous system is impeded by music whereby, music has the capacity to flood the physiological feedback signals which are associated with physical exertion and are of limited processing capacity (Rejeski, 1985; Karageorghis and Priest, 2012a). It is thought that music, through the facilitation of pleasurable stimuli to sensory pathways in the brain, may alter the perception and interpretation of fatigue and hence distract or block sensations of pain and fatigue from focal awareness (Karageorghis and Priest, 2012a). This mechanism can explain the impact of music on low to moderate intensity exercise as individuals are better able to redirect attention to task-irrelevant cues promoting positive affect. However, as the intensity of exercise increases, attentional focus in forced back toward the internal monitoring of task-related cues, i.e., fatigue (Bigliassi et al., 2016). In the aforementioned study, English et al. (2019) suggested that the facilitative nature of music in their findings may be due to the interference of music with afferent feedback of fatigue and pain caused by the exercise and heat strain. Interestingly, they found that music facilitated performance in this capacity up until the last two time blocks where there was a steady decline in self-selected exercise intensity. This may have been due to the exercise and fatigue overriding the motivational music, and it also may be due to the increased thermal perception associated with the relative heat strain which further could be considered as a cognitive load indicating the initiation of a thermoregulatory behavioral response (i.e., reduced exercise intensity) (Gaoua et al., 2012). In the current study, however, the music trials elicited a greater average power output throughout the 15-min TT, which may be due to music overriding the pain and fatigue of the exercise, but also the hypoxic strain.

In this respect, previous research reporting the impact of music on RPE have resulted in inconsistent findings (Elliott et al., 2005; Karageorghis and Priest, 2012a). It has been suggested that music facilitates greater exertion whilst distracting participants from the impact of this increased work output (Karageorghis and Priest, 2012a) hence maintaining RPE. The results from the current study support this suggestion, that RPE remained largely unchanged due to the mechanism of dissociation, a coping mechanism, which facilitates an individual in blocking out the increased discomfort in hypoxia and normoxia. Similarly, previous research has demonstrated that music enhances mood and motivation during exercise (Tenenbaum et al., 2004; Elliott et al., 2005; Karageorghis and Priest, 2012a). In contrast, the current study found music to have no effect on post-exercise mood or motivation. A possible explanation, however, could be that much of this research has recorded mood and motivation change throughout exercise, whereas the current study monitored change from pre- to post-exercise. Considering music, independently, had no impact on ratings of discomfort, mental effort, RPE, mood, and motivation, these results provide further evidence for the theory of dissociation whereby music maintained subjective scores despite a higher power output (i.e., increased pacing).

### Limitations

The current research was conducted in two different environmental conditions including normoxia (0.209 FiO_2_) and normobaric hypoxia (0.13 FiO_2_). A potential limitation of this research and a future direction to consider is that a higher level of hypoxia was not included in the experimental trials, e.g., >0.13 FiO_2_, which would provide a greater insight into the differential impact of music on physical performance in hypoxia. It has previously been reported that at moderate hypoxia, peripheral fatigue is the primary contributor of the decrease observed in the performance of small muscle mass exercise (Millet et al., 2012; Fan and Kayser, 2016; O’Keeffe et al., 2021). Conversely, in more severe hypoxia, it has been observed that central fatigue is the primary factor impacting performance *via* alterations in cerebral oxygenation (Millet et al., 2012). Incorporating a measure of cerebral oxygenation could therefore facilitate a greater interpretation of the impact of music on cerebral dynamics in hypoxia. Further, another consideration for future research includes investigating the impact of music on physical performance in hypobaric hypoxic conditions, which may yield different results.

This study used an arm bike ergometer to represent self-regulated upper body work in normoxia and hypoxia. This was a relatively unfamiliar task to participants, however, utilizing the arm-bike effectively is not difficult. To use the arm-bike, participants were required to push with one arm and pull with the other. All participants attended a familiarization session which included a practice of the entire physical performance protocol prior to undertaking the main experimental trials.

A limitation of research investigating the impact of self-selected music on performance outcomes, is that it is difficult to completely blind participants to the objectives of the study. In the current study, participants were asked to select songs that would motivate them to exercise harder and/or longer as per the guidelines of the BMRI-2 (Karageorghis et al., 2006). Hence, the issue lies in the potential of participants to alter their behavior in the presence of music. Despite this, due to music having no impact on subjective scores (i.e., RPE, discomfort, mental effort, motivation, and mood) we can conclude that participants were unaware of their greater exertion in relation to their increased power output in the time trial and hence, did not alter their behavior as a result of not being blinded to the music component.

With regards to the subjective ratings of perceived exertion, participants were asked to rate their overall exertion on a whole-body scale, which differs to a localized peripheral rating of perceived exertion. As proposed by Hutchinson et al. (2019), individuals unfamiliar or untrained in upper body exercise such as the arm crank, should provide differentiated scores of RPE, i.e., peripheral RPE (arms) and overall RPE (whole-body) to gain a more accurate indication of the exertion of the participant. In the current study, participants untrained in arm-crank exercise were used and therefore whole body RPE may not have been a true representation of their exertion.

Much debate surrounds the use of the doublet interpolation method as a reliable and valid measure for the quantification of muscle activation. Despite its limitations however, this method is widely used to quantify central and peripheral fatigue in maximal conditions (Shield and Zhou, 2004; Folland and Williams, 2007; Lloyd et al., 2016) and is especially useful when comparing muscle activation within-subjects, in a repeated measures design such as the one incorporated in the current study. This study utilized a 300V, doublet square-wave (with a 10-ms stimulus interval) which have been shown to be a better estimate of muscle activation than a single stimulus (Folland and Williams, 2007). Further, participants were harnessed and strapped into the custom-built neuromuscular rig specific to each individual’s height and limb length to ensure optimal and consistent angular positioning. Each participant’s individual stimulus was determined through a ramped protocol in the familiarization session (i.e., the stimulus strength when a plateau in the mechanical response of the muscle was observed), and the electrode placements subsequently marked for consistent stimulation. Baseline doublets were performed prior to the beginning of the physical performance protocol to ensure the correct placement of the electrodes. However, they were not recorded and used for comparative analysis and therefore a further limitation of this study is that the resting doublets were performed after the time trial, prior to performing the MVC, and hence, may already be in a fatigued state.

Lastly, a major limitation of this study is that it included males only. However, research has demonstrated that females may be more susceptible to different psychological constructs at altitude, where sex has been shown to be an independent predictor of significantly heightened anxiety at altitude (Boos et al., 2018). As this study was focused on investigating music as a mechanism to alleviate performance decrements and further, observe mechanisms facilitating the variation in responses to hypoxic environments, focusing on one gender allowed greater clarity in the first instance. However, research is warranted to investigate the psychological constructs which may help to explain some of the variation observed in physical performance, and further alleviate the performance decrements being observed in altitude amongst females.

## Conclusion

Research investigating methods for mitigating the detrimental impact of hypoxia on physical performance have largely examined physiological aids. Despite hypoxia incurring debilitating psychological impairment, limited research has investigated psychological mechanisms for mitigating the negative impact observed in hypoxia. This research demonstrated that self-selected motivational music increased time trial performance and VA% of the bicep through mechanisms such as enhancing neural drive and diminishing detrimental mental processes, in both normoxia and hypoxia. Further, music had no impact on subjective ratings of RPE, mood, mental effort, motivation, or discomfort. However, in the presence of hypoxia, music reduced ratings of mental effort, breathing discomfort and arm discomfort. Hence, the results from the current study suggest that music was used as a dissociative technique where music blocked the sensations of fatigue and discomfort. Overall, the results confirm that music is a viable intervention method for mitigating the detrimental impact of hypoxia on physical performance.

## Data Availability Statement

The raw data supporting the conclusions of this article will be made available by the authors, without undue reservation.

## Ethics Statement

This study involving human participants was reviewed and approved by the Loughborough University Ethics Committee. The participants provided their written informed consent to participate in this study.

## Author Contributions

KO’K, JD, AL, and SH conceived and designed the research. KO’K and JD conducted the experiment. KO’K analyzed the data with support from AL. KO’K drafted the manuscript. All authors read, revised critically, and approved the manuscript.

## Conflict of Interest

The authors declare that the research was conducted in the absence of any commercial or financial relationships that could be construed as a potential conflict of interest.

## Publisher’s Note

All claims expressed in this article are solely those of the authors and do not necessarily represent those of their affiliated organizations, or those of the publisher, the editors and the reviewers. Any product that may be evaluated in this article, or claim that may be made by its manufacturer, is not guaranteed or endorsed by the publisher.
